# Analysis of Corticosterone and Testosterone Synthesis in Rat Salivary Gland Homogenates

**DOI:** 10.3389/fendo.2019.00479

**Published:** 2019-07-17

**Authors:** Takahiro Ieko, Hirokuni Sasaki, Naoyuki Maeda, Jumpei Fujiki, Hidetomo Iwano, Hiroshi Yokota

**Affiliations:** ^1^Laboratory of Veterinary Biochemistry, Department of Veterinary Medicine, Rakuno Gakuen University, Ebetsu, Japan; ^2^Laboratory of Meat Science and Technology, Department of Food Science and Human Wellness, Rakuno Gakuen University, Ebetsu, Japan

**Keywords:** salivary glands, steroidogenesis, corticosterone, testosterone, steroid, pregnenolone, sulfate, conjugate

## Abstract

Extra-adrenal steroid hormone production has been reported in several tissues, the biological role of which is interesting in terms of hormonal regulation of metabolism, growth, and behavior. In this report, we describe for the first time steroidogenesis in rat salivary glands. Enzyme activities associated with corticosterone and testosterone production were detected in rat salivary glands by LC-MS analysis. In tissue homogenates of rat salivary glands, progesterone was produced enzymatically *in vitro* from pregnenolone in the presence of NADPH and NADH. Deoxycorticosterone was produced from progesterone, corticosterone from deoxycorticosterone, and testosterone from androstenedione (but not pregnenolone from cholesterol) via enzymatic reactions using the same tissue homogenates. Immunoblotting analysis indicated the expression of 11β-hydroxylase (cytochrome P450 11β1; CYP11β1), which mediated the production of corticosterone from deoxycorticosterone. However, CYP family 11 subfamily A member 1 (CYP11A1)-mediated production of pregnenolone from cholesterol was not detected in the salivary glands by immunoblotting using a specific antibody. These results indicate that corticosterone and testosterone are produced from pregnenolone in rat salivary glands. The initial substrate in salivary steroidogenesis and the roles of salivary corticosterone and testosterone are discussed.

## Introduction

**Figure 1:**
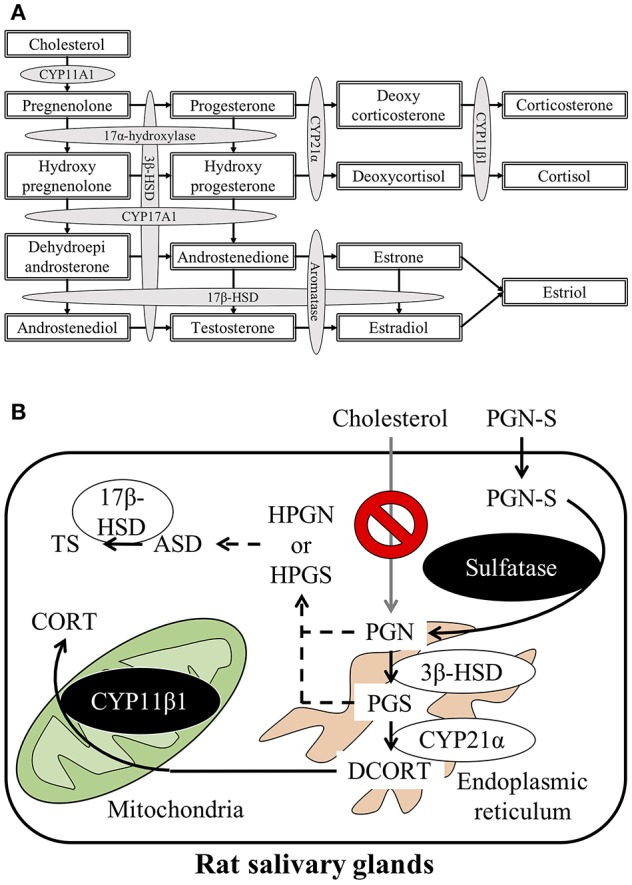
**(A)** Schematic representation of the steroidogenesis pathway. Steroidogenesis involves cytochrome P450 reactions and hydroxysteroid dehydrogenase reactions. **(B)** Schematic illustration of the steroid synthesis pathway in rat salivary glands. Pregnenolone sulfate (PGN-S) in the blood is taken up by the salivary glands and then converted to pregnenolone (PGN) by the enzyme sulfatase. De-conjugated PGN functions as a precursor for the local corticosterone (CORT) or testosterone (TS) synthesis pathways.

Steroid hormones are produced and secreted by endocrine glands such as the gonads, adrenal cortex, and the placenta and act on target organs via the blood. Cholesterol, the first substrate in steroid hormone synthesis, is progressively modified by enzymes located in the mitochondria and smooth endoplasmic reticulum in these organs. Five different cytochrome P450 (CYP) enzymes and two hydroxysteroid dehydrogenases (HSDs) participate in the steroid hormone synthesis process. The principal steroidogenesis pathway is shown in Figure 1A. In the first stage of steroidogenesis, intracellular cholesterol is transported via steroidogenic acute regulatory protein (StAR) and peripheral type benzodiazepine receptor to the mitochondrial inner membrane, where it is converted to pregnenolone (PGN) by CYP family 11 subfamily A member 1 (CYP11A1), which is located on the inner membrane (1). PGN serves as the substrate for subsequent steps in the steroid hormone synthesis pathway. The rate-determining step of steroidogenesis is the transport of cholesterol from the mitochondrial outer membrane to the inner membrane via StAR (2).

After synthesis of deoxycorticosterone (DCORT) by 21α-hydroxylase (cytochrome P450 21α; CYP21α) from progesterone (PGS), the synthesis of corticosterone (CORT) is catalyzed by 11β-hydroxylase (cytochrome P450 11β; CYP11β) located in the mitochondria. Cortisol is synthesized by CYP11β1 from deoxycortisol, which is synthesized by CYP21α from hydroxyprogesterone (HPGS) (Figure 1A). The primary glucocorticoid synthesized in the human adrenal cortex is cortisol, but in rodents, including rats and mice, CORT is the primary glucocorticoid in the adrenal cortex due to the lack of CYP17α (3–5). It was once thought that steroid hormones were synthesized only by endocrine glands, such as the adrenals or testes. However, recent studies have described steroidogenesis in tissues other than endocrine organs (6, 7). For example, mRNAs, proteins, and enzyme activities associated with glucocorticoid synthesis have been described in murine thymic epithelial cells (8, 9). In addition to thymic epithelial cells, thymocytes are also known to express StAR, CYP11A1, 3β-HSD, CYP17, CYP21, and CYP11B1 mRNA and synthesize CORT (10, 11). Locally synthesized glucocorticoids in the thymus exert major effects on thymocyte development and growth (12). Expression of mRNAs and proteins involved in glucocorticoid synthesis has also been reported in mouse small intestine, colon, and murine intestinal epithelial cell lines (13–15). In humans, CYP11A1 and CYP11B2 mRNAs have been detected in the colon, suggesting that glucocorticoid synthesis is possible in the human intestine, as observed in mice (14). Furthermore, glucocorticoids synthesized by intestinal epithelial cells are known to promote epithelial cell proliferation and ameliorate the damage associated with inflammatory bowel disease in both mice and humans (14, 16). Enzymes involved in steroidogenesis are present in the skin also, suggesting that the skin may synthesize glucocorticoids. Glucocorticoid synthesis in the skin is thought to function as a rapid local stress response system (17). Steroids that play important roles in central nervous system development and function are also reportedly synthesized in the brain. Although these brain-derived steroids (i.e., “neurosteroids”) have been extensively studied, the synthesis of sex steroids in the central nervous system in particular has been extensively investigated (18–22). Brain-derived aldosterone is known to play important roles in the regulation of blood pressure and development of high blood pressure (23). Expression of enzymes involved in steroidogenesis has also been detected in human vascular cells and the heart, suggesting local steroid synthesis in the cardiovascular system (24, 25). In addition to these examples, CORT is also reportedly synthesized in the testis and mouse clonal myoblastic C2C12 cells (26, 27). Although numerous reports describe local steroid synthesis in a variety of organs, local steroid synthesis in the salivary gland has not been reported to date.

Saliva plays an important role in maintaining oral homeostasis. In addition to digestive enzymes such as amylase, saliva contains glucocorticoids and sex steroid hormones. In humans, the concentration of salivary CORT is positively correlated (*r* = 0.893) with plasma CORT levels (28, 29). As collection is non-invasive and convenient, it is clinically useful to use saliva as a substitute for blood in determining CORT and cortisol levels for diagnostic purposes (30). Measurement of CORT and cortisol levels in saliva is considered superior for diagnostic purposes because it avoids potential artifacts associated with blood draw stress. However, the concentration of CORT in the blood does not absolutely correlate with the concentration in saliva (31). The significance of salivary CORT is thus unknown. As an initial step in elucidating the significance of salivary CORT, therefore, the present study provides the first report of enzymatic activities associated with salivary steroidogenesis in rats.

## Materials and Methods

### Chemicals and Reagents

The following were purchased from Sigma-Aldrich (St. Louis, MO, USA): precursors of CORT, including PGN, PGS, DCORT; precursor of testosterone (TS), androstenedione (ASD); and conjugated metabolites, pregnenolone-sulfate (PGN-S). Cholesterol was purchased from Wako Pure Chemical Industries (Osaka, Japan). LC-MS–grade acetonitrile and formic acid were purchased from Supelco (Bellefonte, PA, USA).

### Ethics Statement

This study was carried out in strict accordance with the recommendations in the Guide for the Care and Use of Laboratory Animals of the U.S. National Institutes of Health. The protocol was approved by the Committee on the Ethics of Animal Experiments of the Rakuno Gakuen University (permit number: VH17A4). All surgeries were performed under isoflurane anesthesia, and all efforts were made to minimize animal suffering.

### Preparation of Rat Organs

Male (300–400 g) or pregnant female (240–300 g at gestation day 12–13) Sprague-Dawley rats (8 to 15 weeks-old) were purchased from Sankyo Lab Co. (Tokyo, Japan). The rats were fed, housed, and allowed to adapt to their environment for 1 week before the experiments. The salivary glands (SGs), adrenal glands (Adrs), and testes were collected from the male animals, and placentas were collected from the pregnant females, after euthanasia by exsanguination under isoflurane anesthesia. After dissection, the organs were excised post-mortem and then weighed. SGs used for enzyme reaction assays included the sublingual glands (SLGs), submandibular glands (SMGs), and parotid glands (PGs). For the Western blot analyses, the PGs were isolated and used separately as PG and SLG+SMG extracts. A total of 11 male rats were used for enzyme reaction assays (6 animals) and Western blot analyses (5 animals), and a total 4 pregnant female rats were used for enzyme reaction assays or Western blot analyses.

### Antibodies

For Western blot analyses, the following antibodies were used: rabbit polyclonal anti-GAPDH (sc-25778, Santa Cruz), anti-CYP11A1 (ab175408, Abcam), anti-StAR (sc-25806, Santa Cruz), anti-steroid sulfatase (bs-3857R, Bioss), and anti-CYP11β1 (provided by Dr. Mukai) as primary antibodies; and goat anti-rabbit IgG (H+L) horseradish peroxidase conjugate (#1706515, BIO-RAD) was used as the secondary antibody. Anti-CYP11β1 was raised in rabbits by immunization using the peptide corresponding to amino acid residues 272-283 (KNVYRELAEGRQC). For affinity preparation of the antibody, the sulfhydryl group of the carboxy terminal cysteine residue was conjugated with carrier protein (32).

### Determination of Steroid Levels in the SGs

Samples were prepared for MS analysis of steroids as previously described (33). LC-TOF MS peaks corresponding to DCORT and CORT were identified using the SigmaFit™ algorithm (Bruker Daltonics, Bremen, Germany), and other steroids were identified as reported elsewhere (33). For quantification of steroid hormones, a Xevo TQ-S micro triple-stage quadrupole mass spectrometer connected to an Acquity UPLC (Waters, Manchester, UK) and an ESI source device was constructed (LC-MS/MS), as described (33). Steroid hormones were used as standards according to the addition method (29). The limit of detection of PGN, PGS, TS, DCORT, and CORT was 14.2, 3.2, 0.6, 9.2, and 0.4 fmol/g of tissue, respectively, as shown by Maeda (33). The stable isotope corticosterone-d8 was purchased from Otsuka Pharmaceutical (Tokyo, Japan); testosterone-d3 was purchased from Merck (Kenilworth, NJ, USA).

### Assays of Enzyme Reactions

To assay enzymatic activities associated with steroidogenesis, SGs, Adrs, testes, and placentas were washed with phosphate-buffered saline, and then the excised tissue was homogenized with 4 volumes of 0.25 M sucrose and centrifuged at 900 × *g*. The resultant supernatants were used as enzyme preparations. Enzyme activities were assayed as described by Wang et al. (29), with several modifications for the determination of reaction products using an MS analysis method that we developed (33). SG, Adr, testis, and placenta homogenate preparations (each *n* = 3) were incubated with 30 μM each enzyme substrate, cholesterol, PGN, PGS, DCORT, and ASD in 20 mM Tris-HCl buffer (pH 7.4) containing 4 mM MgCl_2_. The reaction was initiated by the addition of 100 μM NADPH and NADH and incubated at 37°C for 2 h. The mixture was then boiled to halt enzyme reactions and vortexed thoroughly. Steroids (final concentration, 5.0 ng/mL) as internal standards were dissolved in 100 μL of acetonitrile containing 0.1% formic acid and added to the boiled mixture, which was then centrifuged at 10,000 × *g* for 10 min at 4°C. Next, 100 μL of hexane was added to the supernatant, and each tube was shaken for 5 min at high speed. After centrifugation, the supernatants (10 μL each) were used for MS analysis (34).

### Western Blot Analysis

For Western blot analysis, samples were immediately frozen with liquid nitrogen and stored at −25°C. The SG (SLG + SMG and PG), Adr, and placenta homogenates (each *n* = 3) were suspended in 0.25 M sucrose solution (200 μL) and then centrifuged at 900 × *g* for 10 min at 4°C. After protein concentrations were estimated using the Lowry protein assay (35), the protein concentration of each sample was adjusted to 1 μg/μL and 20 μg total protein samples were applied. The homogenates were separated by SDS-PAGE and then transferred onto PVDF membranes (ATTO, Tokyo, Japan) for Western blot analysis. After blocking with 3% skim milk at room temperature for 1 h, the PVDF membranes were incubated for 2 h with the primary antibodies (anti-GAPDH, 1:400; anti-CYP11A1, 1:400; anti-StAR, 1:200; anti-steroid sulfatase, 1:200 and anti-CYP11β1, 1:500) at room temperature. After four washes with PBS for 15 min each, the membranes were incubated with secondary goat anti-rabbit IgG (1:2,000) at room temperature for 2 h. After four washes with PBS for 15 min each, the membranes were reacted for 1 min with ECL^TM^ Western blotting detection reagents (Amersham Biosciences, England). The expression level of each protein was determined densitometrically using CS Analyzer, ver. 3.0 (ATTO).

### Statistical Analysis

Results are expressed as the mean ± S.D. of three independent experiments. Statistical significance was assessed using the Mann-Whitney *U* test.

## Results

### Enzymatic Activities Associated With Steroidogenesis in Rat Salivary Glands

**Figure 2:**
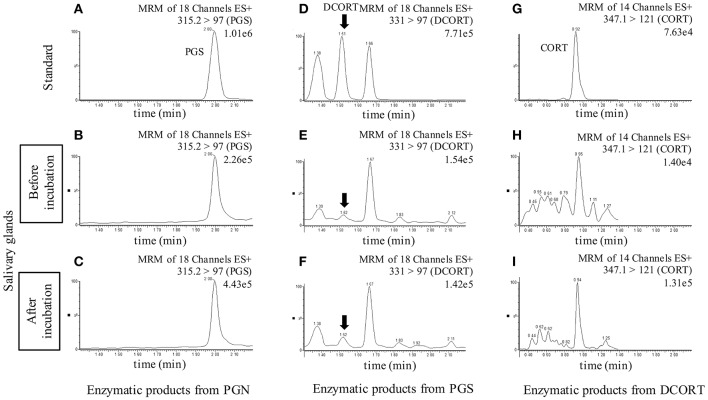
LC-MS analysis of enzymatic products. Chromatograms of standards and exogenous steroids in salivary glands are shown. Values to the upper right of the chromatograms indicate the precursor ion, product ion, and most intense peak (set at 100%). Top: Panels **(A,D,G)** are showing the chromatograms of the standards of each steroid (10 ng/mL), progesterone (PGS), deoxycorticosterone (DCORT), and corticosterone (CORT), respectively. Middle: Panels **(B,E,H)** are showing the chromatograms of the incubation mixtures added with pregnenolone (PGN), PGS, and DCORT before incubation, respectively. Bottom: Panels **(C,F,I)** are showing the chromatograms of the reaction mixtures added with PGN, PGS, and DCORT after a 2-h incubation, respectively. In the DCORT chromatogram, multiple peaks were confirmed, and black arrows indicate DCORT. MRM, multiple reaction monitoring. The intensity of the CORT peak with a retention time of 0.92-0.95 min after the enzyme reaction (I, 1.31e5) was 9.36-fold higher than that of the peak before incubation (H, 1.40e4). Each steroid was analyzed three times in each sample, and representative results are shown.

To investigate the synthesis of CORT in rat SGs, we carried out enzymatic reactions associated with steroidogenesis using tissue homogenates of rat SG (mixtures of SMG, SLG, and PG) and homogenates of rat Adr as a positive control. The reaction products were analyzed by LC-MS, and we observed strong peaks indicative of the enzymatic production of PGS from PGN, DCORT from PGS, and CORT from DCORT (Figure 2). These data suggest that the rat SG homogenate contained enzymatic activities associated with the synthesis of CORT from PGN.

**Figure 3:**
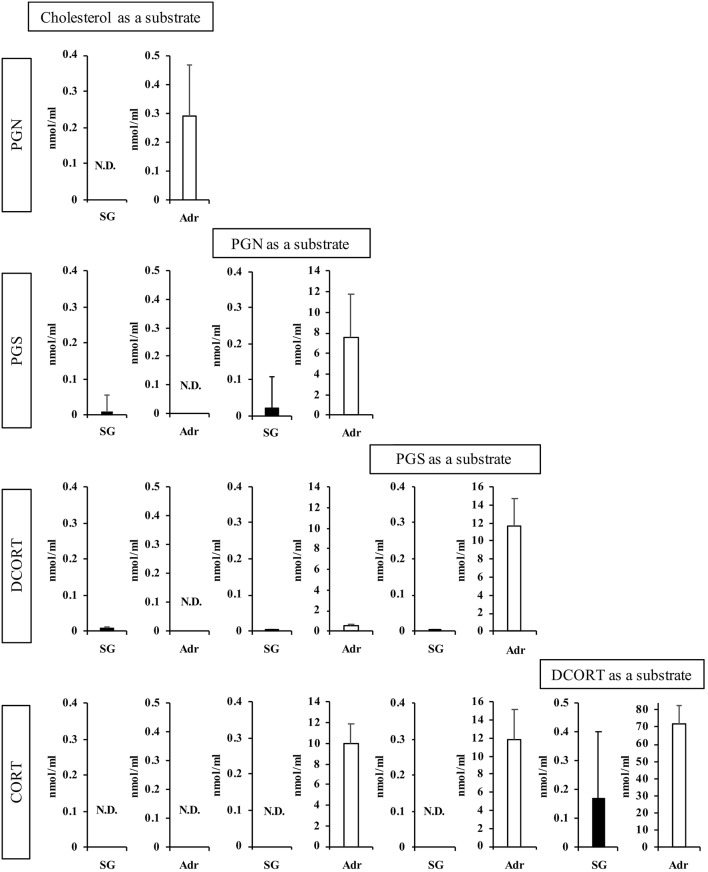
Enzymatic activities associated with steroidogenesis in rat salivary gland (SG) and adrenal gland (Adr). The concentrations of pregnenolone (PGN), progesterone (PGS), deoxycorticosterone (DCORT), and corticosterone (CORT) produced from each substrate (cholesterol, PGN, PGS, and DCORT) were determined after a 2-h enzymatic reaction using rat SG (*n* = 3, closed bars) and Adr (*n* = 3, open bars) homogenates and LC-MS analysis. Data are shown as the mean ± S.D.

Next, we determined the concentrations of enzymatic reaction products after 2 h (Figure 3). Using PGN and PGS as substrates, the production of subsequent steroid hormones was observed in the rat SG homogenate, although the concentrations were lower compared with the Adr. Interestingly, the level of CORT produced in the rat SG homogenate was higher compared with other steroids synthesized in the tissue. These findings indicated that the CORT synthesis pathway is functional in rat SGs. PGN was produced from cholesterol in reactions using the Adr homogenate. As shown in Figure 3, however, PGN was not produced from cholesterol in the rat SG homogenate.

**Figure 4:**
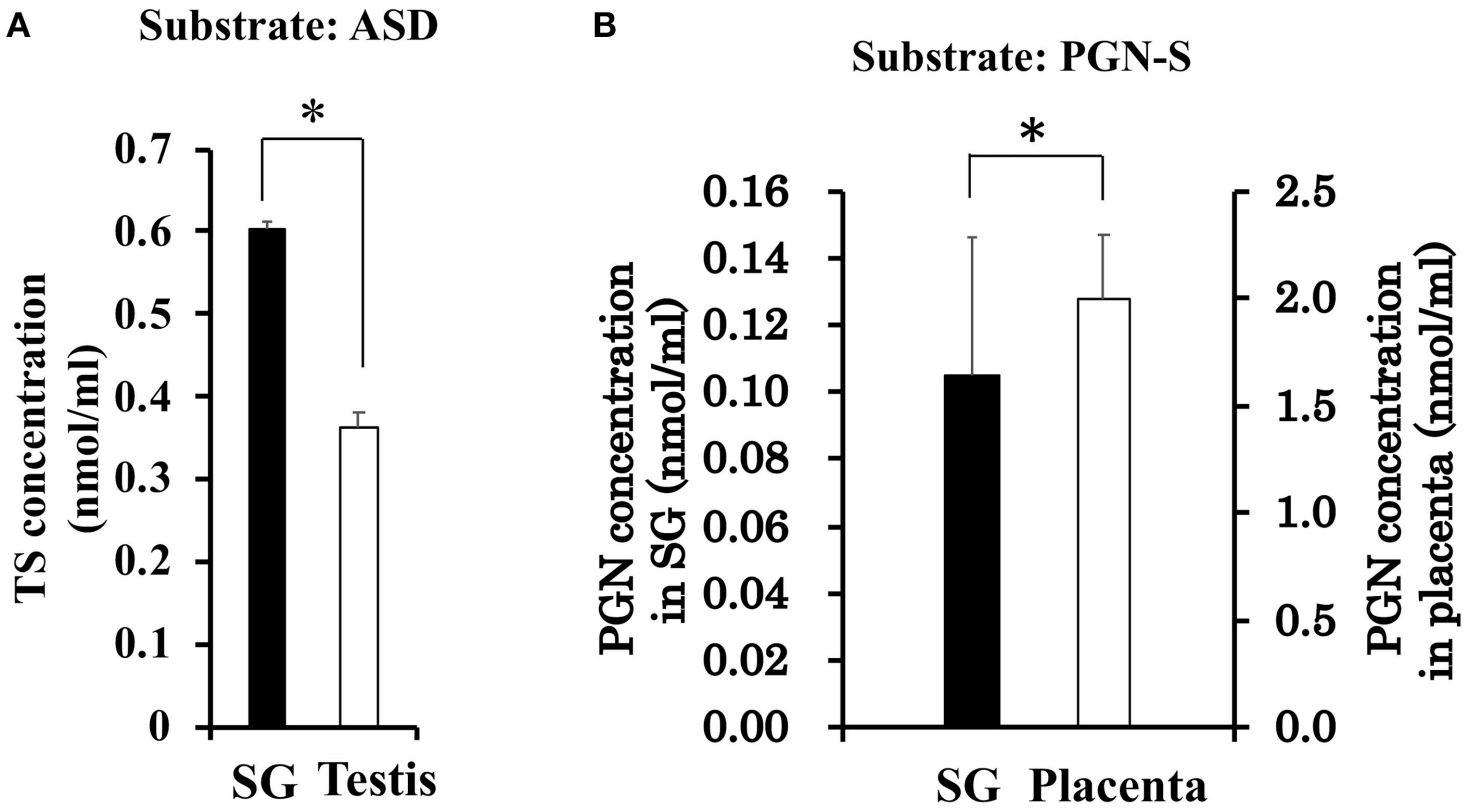
**(A)** Enzymatic activity associated with testosterone (TS) synthesis in rat salivary gland (SG) and rat testis. The concentration of TS produced from androstenedione (ASD) was determined by LC-MS analysis after a 2-h enzymatic reaction using homogenates of rat SG (*n* = 3, closed bars) and rat testis (*n* = 3, open bars). **(B)** Enzymatic activity associated with sulfate de-conjugation in rat salivary gland (SG) and rat placenta. The concentration of pregnenolone (PGN) produced from pregnenolone sulfate (PGN-S) in rat SG (*n* = 3, open bars) and rat placenta (*n* = 3, closed bars). Data are shown as the mean ± S.D. ^*^p < 0.05.

With the interest of characterizing not only the CORT synthesis pathway but also the potential synthesis of TS in rat SGs, we also examined the synthesis of TS from ASD in the rat SG homogenate and positive control rat testis homogenate. Interestingly, the level of TS synthesized in rat SGs was the same as that synthesized in the testis (Figure 4A).

### Expression of Steroidogenesis-Associated Enzymes in Rat Salivary Glands

**Figure 5:**
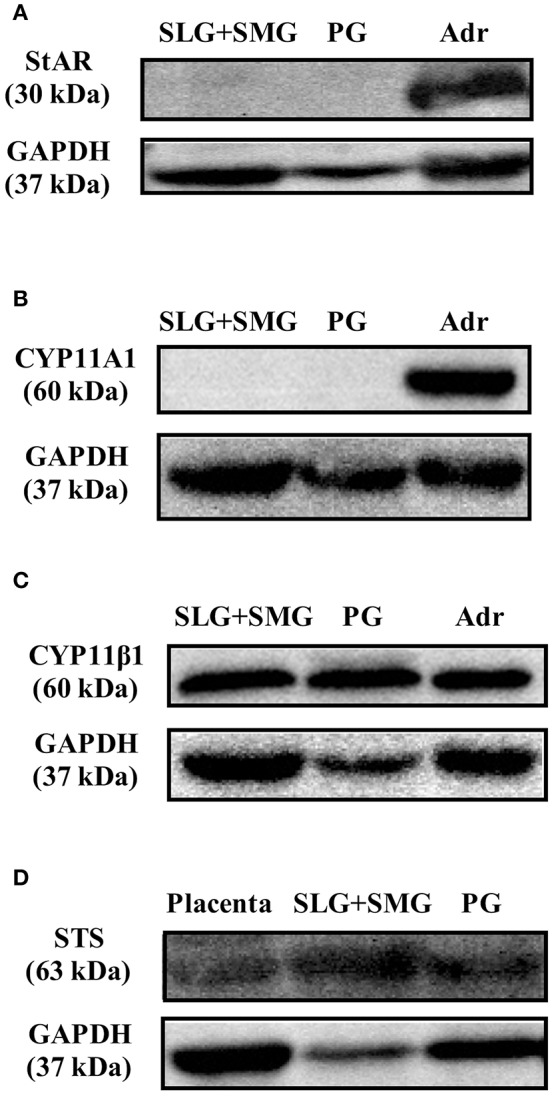
Western blotting analysis of **(A)** steroidogenic acute regulatory (StAR) protein expression, **(B)** CYP family 11 subfamily A member 1 (CYP11A1) protein expression, and **(C)** cytochrome P450 11β (CYP11β1) protein expression in rat sublingual gland and submandibular gland (SLG+SMG), parotid gland (PG), and adrenal gland (Adr) using respective antibodies. **(D)** Western blotting analysis of steroid sulfatase (STS) protein expression in rat SLG+SMG, PG, and placenta using anti–steroid sulfatase antibody. Each protein was analyzed three times, and representative results are shown.

With the exception of enzymatic activity associated with the production of PGN from cholesterol, activities associated with steroidogenesis were observed in rat SGs. We examined whether the enzymes involved in PGN production are expressed in rat SGs using Western blotting analysis with specific antibodies. Using tissue homogenates of rat SLG + SMG, rat PG, and rat Adr as a positive control, we did not detect StAR, a transport protein that regulates cholesterol transfer within mitochondria, or CYP11A1, the enzyme that cleaves the cholesterol side chain to produce PGN (Figures 5A,B). These results demonstrated that although CORT and TS are produced from PGN in rat SGs, cholesterol does not serve as the primary steroidogenesis substrate.

We also examined the expression of CYP11β1, the enzyme that produces CORT from DCORT. High levels of CYP11β1 expression were observed in rat SLG + SMG and PG homogenates (Figure 5C). This result was in agreement with the results demonstrating high levels of CORT production in tissue homogenates (Figure 3).

### Activity and Expression of Enzymes Associated With De-conjugation in Rat SGs

To examine the de-conjugation of PGN-S in rat SGs, we carried out enzymatic reactions and Western blotting analysis using rat SG tissue homogenates, with placenta homogenate serving as a positive control. We detected PGN, the de-conjugation product of PGN-S, in rat SG homogenate (Figure 4B). We then performed Western blotting analysis of the expression of steroid sulfatase (STS), the enzyme that de-conjugates sulfated steroid hormones, using homogenates of rat SLG+SMG, PG, and placenta. STS was detected in the rat SLG+SMG and PG homogenates at levels similar to that observed in the placenta (Figure 5D).

## Discussion

Saliva is produced by the SGs and secreted intraorally. Three major types of SGs have been described: SMGs, SLGs, and PGs. In this study, we detected enzymatic activities and the expression of two principal enzymes that mediate the production of CORT in homogenates of rat SGs. These results indicate that CORT and TS are synthesized in rat SGs. We could not detect the expression of CYP11A1, however, which led us to attempt to identify the primary substrate of steroid synthesis in rat SGs. Dehydroepiandrosterone (DHEA)-sulfate is produced in the adrenal cortex and transported via the blood to peripheral tissues such as the placenta, where it is de-conjugated to form DHEA and serves as the substrate for TS and estrogen production (36, 37). We initially reported that PGN-S is the primary substrate for steroidogenesis in mouse clonal myoblastic C2C12 cells (27). Because PGN-S is abundant in rat blood (33), we postulated that PGN-S serves as the primary substrate for steroidogenesis in SGs as well. Our results confirmed the presence of sulfatase enzymatic activity and expression of the sulfatase enzyme in the same homogenates of rat SGs. In particular, our results suggest that rat SGs produce PGN from PGN-S and use it as the initial substrate for steroidogenesis.

The results of our study suggest that steroid hormones are synthesized in tissues throughout the body. Previous studies have reported extra-adrenal or extra-gonadal steroid hormone production. In particular, sex steroid hormones are reportedly produced in the brain hippocampus, where they exert many local physiologic effects on nerve cells (19, 38, 39). Other reports have demonstrated the synthesis of cortisol and aldosterone in the brain (22, 40), androgen and estrogen synthesis in the liver, and glucocorticoid synthesis in the intestinal tract and skin (6, 22). In addition to these reports, we show the possibility of steroidogenesis in the SGs for the first report in this study, indicating that steroid hormones are synthesized in tissues throughout the body, including the SGs.

SGs have the same levels of enzyme activity of TS production as that in the testis. A significant amount of TS could be secreted into saliva. Salivary TS may regulate not only the oral cavity tissue homeostasis, but also the digestive tract functions. In addition, since the enzyme activity value of CORT in SGs is about 0.23% as compared with that in adrenal glands and the intermediate products PGN and DCORT have low synthetic activities in the SGs, it is considered that the final product CORT synthesized by SGs is less synthetic amount. Therefore, it is considered that the secretion of salivary CORT to saliva is also little amount. In this case, salivary CORT may act as a paracrine or autocrine factor in the glands. As SG cells and oral cavity tissues express receptors for steroid hormones (41–43), CORT and TS may act through these receptors in these putative target tissues. They may affect the activities in the site specific cells in the SGs and then regulate the component fluctuation of saliva as reported in the intestine (14, 16) and in the skin (17). The paracrine effects of cytokines, myokines, and adipokines secreted by immune cells, muscle cells, and adipocytes, respectively, have attracted considerable recent research attention (44–46). These factors are also involved in autocrine metabolic regulation of secretory tissues (44, 46). It is thought that the steroids synthesized throughout the body work together with cytokines, myokines, and adipokines via cross-talks between tissues (47) or as metabolic regulators within specific tissues (19).

Amylase secretion via a protein kinase A–independent cAMP pathway has also been reported (48, 49). Glucocorticoids such as CORT are secreted in response to stress, leading to increased blood glucose levels via gluconeogenesis in the liver. The glucocorticoids inhibit the uptake of glucose by competing with the insulin response in skeletal muscle and white adipocytes (50, 51). As a result of the stress-induced increase in blood glucose levels, sympathetic nerves are activated by local synthesis of glucocorticoids in the SGs, and β-receptors are stimulated by the release of noradrenaline. It has been reported that blood levels of both cortisol and α-amylase are higher in patients with depression than in non-depressed patients (2). Accordingly, glucocorticoids synthesized locally in the SGs could exert physiologic effects such as regulating the secretion of digestive enzymes in the tissues.

The results of the present study suggest that CORT is synthesized in rat SGs using PGN-S as the primary substrate instead of cholesterol (Figure 1B). How steroids synthesized locally in the SGs regulate secretion by the SGs is an interesting topic for research into the biological role of these glands. In addition, TS is synthesized in significant amounts from ASD in rat SGs. The regulation and biological importance of TS secreted into the saliva also represent interesting topics for future research.

Human salivary glands are composed with parotid, sublingual and submandibular glands as that in rat (52). It is considerable that local steroidogenesis is performed in human salivary glands too. By the detailed investigation about human salivary steroidogenesis and its roles, we will obtain some endocrinological new evidences.

## Data Availability

The datasets for this study will not be made publicly available because Principal of our manuscript does not need any datasets.

## Ethics Statement

This study was carried out in strict accordance with the recommendations in the Guide for the Care and Use of Laboratory Animals of the U.S. National Institutes of Health. The protocol was approved by the Committee on the Ethics of Animal Experiments of the Rakuno Gakuen University (permit number: VH17A4). All surgeries were performed under isoflurane anesthesia, and all efforts were made to minimize animal suffering.

## Author Contributions

TI, HS, and HY conceived the experiments. TI, HS, and JF carried out the experiments. TI, HS, and HY analyzed the data and wrote the manuscript. NM supervised the LC-MS analyses. HI and HY supervised the project.

### Conflict of Interest Statement

The authors declare that the research was conducted in the absence of any commercial or financial relationships that could be construed as a potential conflict of interest.

## Abbreviations

YP: cytochrome P450
CYP11β: 11β-hydroxylase (cytochrome P450 11β)
CYP11A1: CYP family 11 subfamily A member 1
SMG: submandibular gland
SLG: sublingual gland
PG: parotid gland
HSD: hydroxysteroid dehydrogenase
StAR: steroidogenic acute regulatory protein
PGN: pregnenolone
PGS: progesterone
HPGS: hydroxyprogesterone
DHEA: dehydroepiandrosterone
ASD: androstenedione
TS: testosterone
CORT: corticosterone
DCORT: deoxycorticosterone
CYP21α: 21α-hydroxylase (cytochrome P450 21α)
Adr: adrenal gland
STS: steroid sulfatase.
